# Modelling the role of microbial p-cresol in colorectal genotoxicity

**DOI:** 10.1080/19490976.2018.1534514

**Published:** 2018-10-25

**Authors:** Eiman Abdulla Al Hinai, Piyarach Kullamethee, Ian R. Rowland, Jonathan Swann, Gemma E. Walton, Daniel M. Commane

**Affiliations:** a Department of Food and Nutritional Sciences, University of Reading, Reading, UK; b Dietetics Department, Al Nahdha Hospital, Ministry of Health, Muscat, Sultanate of Oman; c Department of innovation and technology of product development, Faculty of Agro-industry, King Mongkut’s University of Technology North Bangkok, Prachinburi, Thailand; d Faculty of Medicine, Department of Surgery & Cancer, Imperial College London, London, UK; e Department of Applied and Health Sciences, University of Northumbria, Newcastle Upon Tyne, UK

**Keywords:** p-cresol, tyrosine, colonic fermentation, microbiota, genotoxicity, gut fermentation model, colorectal cancer

## Abstract

**Background**: A greater understanding of mechanisms explaining the interactions between diet and the gut microbiota in colorectal cancer is desirable. Genotoxic microbial metabolites present in the colon may be implicated in carcinogenesis and potentially influenced by diet.

**Aims**: We hypothesised that microbial p-cresol is a colonic genotoxin and set out to model potential exposures in the colon and the effects of these exposures on colonic cells.

**Methods**: Batch culture fermentations with human faecal inoculate were used to determine the synthesis of p-cresol and other metabolites in response to various substrates. The fermentation supernatants were evaluated for genotoxicity and the independent effects of p-cresol on colonic cells were studied *in vitro*.

**Results**: In batch culture fermentation, supplementary protein increased the synthesis of phenols, indoles and p-cresol, whereas supplementary fructoligosaccharide (FOS) increased the synthesis of short chain fatty acids. The p-cresol was the greatest predictor of genotoxicity against colonocytes in the fermentation supernatants. Spiking fermentation supernatants with exogenous p-cresol further increased DNA damage, and independently p-cresol induced DNA damage in a dose-dependent manner against HT29 and Caco-2 cells and influenced cell cycle kinetics.

**Conclusions**: In the colon p-cresol may reach physiologically significant concentrations which contribute to genotoxic exposures in the intestinal lumen, p-cresol production may be attenuated by substrate, and therefore diet, making it a potential modifiable biomarker of genotoxicity in the colon.

## Introduction

The colon is the most common site for intestinal tumours^1^ with microbial activity being implicated in increased susceptibility to neoplastic transformation.^2^ Environmental factors, particularly diet, modulate the composition and metabolic activity of the colonic microbiota with implications for cancer risk.^3,4^ Current mechanistic models implicating diet in CRC risk propose that dietary fibre favourably improves the balance of the microbiota, increasing the abundance of saccharolytic species relative to proteolytic microbes. The latter are associated with increased production of an assortment of genotoxic metabolites from meat based or endogenous substrates.^4–6^ Epidemiological studies implicate red and processed meat in particular in increasing risk of CRC. Genotoxicity associated with haem, *N*-nitroso compounds, and heterocyclic amines has been proposed as a mechanism underpinning this association.^7^ Amongst proteolytic metabolites present in the colon, p-cresol is a relatively poorly studied potential contributor to the genotoxic load.^8^ p-cresol is a methyl phenol produced via microbial degradation of tyrosine.^9,10^ *In situ*, it is absorbed and metabolised in the liver, producing p-cresol sulphate, which is excreted in the urine. Elevated urinary p-cresol sulphate has been observed in patients with colorectal cancer^10^, it may be associated with ageing^11^ and more recently it has been suggested as a biomarker of protein intake.^12^

Due to the efficient intestinal uptake of p-cresol, and other colonic luminal genotoxins, the genotoxicity and chemical composition of faecal samples may be poorly representative of colonic exposures; this has, in part, limited the use of faecal water genotoxicity as a diet-related biomarker of colorectal cancer risk.^13^ The presence of p-cresol sulphate in urine and its association with both diet and the microbiome may make it a useful, modifiable, diet-sensitive, biomarker of colorectal genotoxicity, and therefore potentially of CRC risk, which could be applied in human intervention studies.^12^

Here our objectives were two fold, a) to establish the potential luminal exposure to p-cresol using a simulated gut fermentation system. b) to determine the genotoxicity of p-cresol, as part of the colonic metabolome, at levels of exposure consistent with those achievable *in vivo* using two separate cell-based models of the colonic epithelium.

## Materials and methods

### Chemicals

p-cresol, agarose, EDTA, Trizma base, Triton X-100, hydrogen peroxide (H_2_O_2_), Hepes, ethidium bromide, propidium iodide (PI) and RNase A were purchased from Sigma-Aldrich Ltd. (Dorset UK). Sodium chloride (NaCl) and potassium chloride (KCl) were supplied by Fisher Scientific **(Loughborough, UK)**.

Tyrosine, fructoligosaccharide (Raftilose P95), albumin, soybean protein peptone meat extracts were all purchased from Sigma-Aldrich Ltd. (Dorset UK). Bacteriological growth medium supplements were obtained from Oxoid Ltd. (Basingstoke, Hants, U.K.). Probes for fluorescence *in situ* hybridisation were commercially synthesised and labelled at the 5′ end with the fluorescent dye Cy3 (Sigma Aldrich Ltd, Poole, Dorset, UK). The probes used are listed in Table 1: The HT29 and Caco-2 human colorectal cell lines were obtained from the European Collection of Animal Cell Cultures (ECACC) (Salisbury, UK)

**Table 1.** Primer sequences for fluorescent in situ hybridisation.

Probes name	Sequences 5′ To 3’	Target genus	Reference
Non Eub (Negative control)	ACTCCTACGGGAGGCAGC		^14^
Eub338 I + (Positive control)	GCT GCC TCC CGT AGG AGT	Most bacteria	^15^
Eub338 II +	GCA GCC ACC CGT AGG TGT	Planctomycetales	^15^
Eub338 III +	GCT GCC ACC CGT AGG TGT	Verrucomicrobiales	^15^
Bif164	CATCCGGCATTACCACCC	Most *Bifidobacterium* spp	^16^
Lab158	GGTATTAGCAYCTGTTTCCA	Most *Lactobacillus, Leuconostoc* and *Weissella* spp	^17^
Bac 303	CCAATGTGGGGGACCTT	Most Bacteroidaceae and Prevotellaceae, some Porphyromonadaceae	^18^
Erec 482	GCTTCTTAGTCARGTACCG	Most of the *Clostridium coccoides-Eubacterium rectale* group	^19^
Rrec 584	TCAGACTTGCCGYACCGC	*Roseburia* subcluster	^20^
Chis150	TTATGCGGTATTAATCTYCCTTT	Most of the *Clostridium histolyticum* group *(Clostridium clusters I and II)*	^19^
Prop 853	ATTGCGTTAACTCCGGCAC	*Clostridial* cluster IX	^20^
Ato 291	GGTCGGTCTCTCAACCC	*Atopobium, Colinsella, Olsenella* and *Eggerthella* spp.	^21^
Fprau 647	CGCCTACCTCTGCACTAC	*Faecalibacterium prausnitzii* and related sequences	^22^
DSV 687	TACGGATTTCACTCC T	Most *Desulfovibrionales* (excluding *Lawsonia)* and many *Desulfuromonales*	^23^

Essential Medium (MEM), McCoy’s 5A with L-glutamate, Penicillin-Streptomycin and Fetal Bovine Serum (FBS) were purchased from Biosera Ltd. (East Sussex, UK). Phosphate Buffered Saline (PBS), Non-essential Amino Acids (NEAA), Trypsin-Versene and Ethylenediaminetetraacetic acid (EDTA) were purchased from Lonza group Ltd. (Basel, Switzerland).

### Faecal inoculate for batch culture fermentation

Faecal samples were collected from three individuals (over 60 years of age). All volunteers self-reported as being healthy, antibiotic-free for at least 6 months prior to sampling and free from gastrointestinal disorders. Samples were collected on the day of the experiment and were used immediately. Upon collection, they were diluted 1:10 (w/v) with anaerobic PBS (0.1 M; pH 7.4) and homogenised in a stomacher for 2 min (460-paddle beats/min). 15 ml of the resulting faecal slurries from each individual were used to inoculate batch culture vessels in triplicate.

### Batch culture fermentation

Batch culture fermentation vessels were autoclaved and filled with 135 ml of basal nutrient medium (peptone water (2 g/L), yeast extract (2 g/L), NaCl (0.1 g/L), K_2_HPO_4_ (0.04 g/L), NaHCO3 (2 g/L), MgSO_4_7H_2_O (0.01 g/L), CaCl_2_6H_2_O (0.01 g/L), Tween 80 (2 ml/L), hemin (50 mg/L), vitamin K1 (10 ml/L), L-cysteine (0.5 g/l), bile salts (0.5 g/L), resazurin (1 mg/L) and distilled water (Sigma, Aldrich, UK). The vessels were gassed overnight with O_2_-free N_2_ (15 ml/min).

#### Supplementary substrates

Alternative additional substrates were included to explore the influence of dietary substrates on p-cresol fermentation. These were prepared as a high tyrosine (supplementary tyrosine at 0.3 % w/w), a low tyrosine treatment (supplementary tyrosine 0.003 % w/w), a high tyrosine with fructoligosaccharide (FOS) (0.3 % w/w Tyr + FOS 1.5% w/w), a low tyrosine with FOS (0.003 % w/w Tyr + 1.5% w/w FOS), an albumin treatment (0.3% w/w), a soybean protein treatment (0.3 % w/w) and a peptone meat extract treatment (0.3 % w/w).

Faecal inoculate (15ml) was added to initiate the cultures and the subsequent fermentation carried out under anaerobic conditions. The temperature was maintained at 37°C by use of a circulating water bath and pH was maintained at 6.8 using a pH controller (Electro lab, UK). At six time points (0, 4, 8, 24, 30 and 48 hours), 10 ml of fermentation supernatant was collected for analysis. Fermentation supernatants were filter sterilised through a 0.2 mm PVDF single use filter (Sartorius Ltd. Surrey UK) for use as microbe-free treatments in cell culture experiments.

All fermentation conditions were conducted in triplicate, each time with a different donor- then to eliminate variability associated with the starting culture, fermentations with inoculate from one single donor were repeated three times.

### Bacterial enumeration

#### *Fluorescence* in situ *hybridisation*

Bacterial populations from the batch culture samples were enumerated using fluorescence *in situ* hybridisation and flow cytometry (Flow-FISH), with oligonucleotide probes targeting specific regions of 16S rRNA as described previously.^14^ Briefly, commercially synthesised probes were coated with the fluorescent dye, Cy3 (Table 1). Bacteria from fermentation samples were isolated via centrifugation, washed in PBS and fixed in 4% (v/v) paraformaldehyde, they were then washed and stored at −20^◦^C in PBS and 99% ethanol (1:1 v/v). For analysis, bacteria were prepared in TE-FISH buffer and incubated with lysozyme (1 mg/mL of 50 000 U/mg protein) for 10 min. The cells were then washed and hybridisation completed by incubating the cells in 150 μL of hybridisation buffer (5 M NaCl, 1 M Tris/HCl pH 8, 30% formamide, ddH2O, 10% SDS and 4.55 ng ml^−1^ probe) for 4 hours.

##### Metabolite characterisation

Standards of p-cresol, phenol and indole were prepared in distilled water at concentrations from (0.1–1600 mM) and standard curves plotted following quantification via solid-phase micro-extraction (SPME) gas chromatography mass spectrometry (GC-MS) using an Agilent 110 PAL injection system and Agilent 7890 gas chromatograph with a 5975C mass spectrometer (Agilent, Santa Clara, CA). The SPME fibre stationary phase was composed of 75 µm divinylbenzene/Carboxen™ on polydimethylsiloxane; Supelco, Bellefonte, PA). Sample (0.1 mL) was placed in a 20-mL headspace vial with magnetic screw cap and PTFE/silicone septum (Supelco). The samples were allowed to equilibrate for 10 minutes at 35 °C before being extracted for 30 min. Sample was agitated at 500 rpm (5 seconds on, 2 seconds off) during equilibration and extraction. After extraction, the contents of the fibre were desorbed onto the front of a Stabilwax-DA fused silica capillary column (30 m 0.25 mm i.e., 0.50 mm film thickness; Restek, Bellefonte PA). The GC temperature program and the fibre desorption step commenced at the same time. During the desorption period (45 s), the oven was held at 40 °C. After desorption, the oven was held at 40 °C for a further 255 s before incremental heating at 4 °C/min to 260 °C, where the temperature was maintained for 5 min. Helium was used as the carrier gas at a constant flow rate of 0.9 mL/min. The mass spectrometer was operated in electron impact mode with an electron energy of 70 eV, scanning from m/z 20 to m/z 280 at 1.9 scans/s.

### Organic acid analysis

Samples from batch culture fermentation were screened for short-chain fatty acid (SCFA) concentrations using the gas chromatography method of Zhao et al.^15^ Briefly the fermentation samples were defrosted, vortexed and centrifuged for 5 minutes at 13400 x g. The samples were filtered using a 0.22 μm filters into a new Eppendorf tube. 100 µl of the sample supernatant was added to 260 µl sulphuric acid solution (20 µl sulphuric acid diluted in 100 ml of water, pH 2). 200 µl of the sample and 50 µl of internal standard were prepared in GC vials. The internal standard was 2-ethylbutyric acid (Aldrich) in HPLC grade water. The GC apparatus was calibrated for detection of acetate, propionate, iso-butyrate, butyrate, iso-valeric, valeric and caproic acid. 260 µl sulphuric acid solution was added to 100 µl (100mM) of each standard. 200 µl of this was removed to a vial and added to 50 µl of the internal standard. This was repeated through a range of concentration between 5–100 mM. A HP 5890 series II GC system (Hewlett Packard, Palo Alto, Calif) with an FFAP, capillary fused silica packed column 25 m by 0.32 mm; filter thickness, 0.25µm (Macherey-Nagel, Düren, Gemany). Afterwards, the sample was injected into the column, which was maintained at 140°C for 5 minutes. Then the column temperature was increased over 5 minutes to 240°C. The calibrated organic acids were detected in the samples and the concentration were calculated. Internal standard with known concentration of SCFA was injected after every 10 samples to maintain appropriate calibration. Finally, peaks were analysed and integrated using HP GC ChemStation Software, Hewlett Packard.

### Cell culture

The HT29 and Caco-2 human cell lines are derived from colonic epithelial adenocarcinoma cells and are widely used in cancer research. The cells were used between passages 45 and 70. HT29 cells were cultured routinely in McCoy’s 5A with L-glutamate supplemented with penicillin-streptomycin and FBS. Caco-2 cells were cultured in MEM supplemented FBS and NEAA. Routine culture was carried out at 37°C with 5% CO_2_ and 95% humidity, the cell medium was changed every 2 days with trypsin mediated passage at 80–90% confluence.

### Cell viability

Cell viability was assessed as described previously.^14^ Briefly, HT29 and Caco-2 cells were seeded at 1 × 10^6^ cells per ml and cultured for 24 hours in basal media (37 °C, 5% CO_2_. A concentration curve of 4 cresol in basal media was established and used to treat cells for a further 24 hours. Media was then removed and cells fixed in ice cold methanol, dried and stained with 4,6-diamidino-2-phenylindole, dihydrochloride (DAPI) (70 µl of DAPI staining stock solution (3 mM) plus 10.43 ml of PBS per plate) Absorption was measured using a GENios microplate reader (TECAN Group Ltd., Männedorf, Switzerland) with absorbance and emission at 340 nm and 465 nm, respectively. Cell viability was determined relative to a 0 % p-cresol control.

### DNA damage

HT29 and Caco-2 cells were seeded at concentrations of 1 × 10^6^ and maintained at 37°C in an atmosphere of 5% CO_2_ and 95% filtered air. Cells were treated in the tissue culture flasks at 80% confluency, with p-cresol at final concentrations of between 0 and 3 uM, or with filter sterilised fermentation supernatants prepared at 10% (v/v), in McCoy’s carrier culture medium with inactivated FBS and antibiotics. The cell cultures were incubated at 37°C with 5% CO2 and after 24 hours, DNA damage was assessed using the Comet assay as described previously.^16^ Briefly, a positive control was prepared with untreated cells exposed to 7.5 mM H_2_O_2_ for 15 minutes prior to lysis. Viable cells were counted after trypan blue staining and adjusted to a concentration of 3 × 10^6^ cells/ml. An aliquot (120ul) of the cell suspension was mixed with 200 µl of melted agarose and bedded on microscope slides. The slides were placed into lysis buffer (2.5M NaCl, 0.1 EDTA, 0.01 M Tris and 1% (v/v) Triton X-100) for 1 hour at 4°C, and then washed 3 times with neutralising buffer (0.4M Trizma base pH 7.5) before transfer into electrophoresis buffer (0.3M NaOH and 1mM EDTA). After 20 min at 4°C the slides were placed horizontally in an electrophoresis tank containing electrophoresis alkaline buffer to allow the DNA to unwind. Electrophoresis was run at 26V, 300mA for 40 minutes in at 4°C in the dark. The slides were then washed with neutralising buffer three times for 5 minutes each and left for 5 minutes in 99% ethanol for 5 minutes, then left to dry overnight. Cells were stained with ethidium bromide (20ul/ml) and kept for 15 minutes in the dark. Images of DNA integrity were captured by fluorescence microscopy using the Kinetic image software, Komet 4.0 UK. One hundred randomly selected cells from each replicate slides were evaluated for DNA tail damage by an analyst blinded to the treatment.

#### Spiked fermentation supernatants

To determine the genotoxic potential of p-cresol in the context of the fermentation milieu, selected filter sterilised fermentation supernatants were spiked with exogenous 4 cresol at concentrations of 0.2 or 3 mM and applied to the HT29 cell line for 24 hours prior to the Comet assay.

### Cell cycle assay

Cell cycle progression was assessed considering the percentage of cells in phases Gap0/1 (*G_0_/G_1_*), Synthesis (*S*), Gap2/mitosis (*G_2_/M*) and apoptotic cells (sub *G_0_/G_1_*) according to the fluorescence intensity of a PI nuclear stain, and based on the concentration of DNA within the cell. HT29 and Caco-2 cell cultures were treated at 2 × 10^5^ cells/well into 6 well plates at 80% confluence. The cells were exposed to p-cresol at 0.2, 0.5, 1.0, 1.5, 3.0 mM for 24 hours. After removing treatments, the cells were washed with ice cold PBS and collected following trypsin harvest of the monolayer and pelleting by centrifugation at 300 G for 3 minutes. The supernatants were discarded and then the cell tissues were resuspended in 200 μl ice cold PBS and fixed with 2 ml of fresh ice cold 70% ethanol. The cell pellets were stored in freezer at −20°C until analysis.

After chilling, the samples were centrifuged at 300 G for 5 minutes and the supernatants discarded. The pellets were resuspended with 200 µl PBS before adding 25 µl of 1 mg/ml RNAse and the suspensions were then incubated at 37°C for 30 minutes. 2.5 μl of 400 µg/ml of PI were added to bind DNA and were left to incubate for 30 min at room temperature in dark condition. Cells suspensions were adjusted to a final volume of 600 µl with PBS. The DNA content of 15,000 cells were then measured immediately via flow cytometry (BD Accuri C6 flow cytometer, Germany). Analysis was performed using the Flow Jo software (Tree star Inc, Oregon, USA).

### Statistical analysis

All statistical analyses were carried out using the Statistical Package for Social Sciences (SPSS) version 22. All experiments were carried in three biological replicates for each analysis and the data presented as mean ± SEM. Cell viability and cell cycle data were analysed using linear regression models. For bacteriology, comparisons between each volunteers were made by ANOVA. Similarly the ANOVA was used to compare the effects of substrates on fermentation sample genotoxicity and cell cycle kinetics. Where appropriate comparison of individual treatments with negative control were performed using the Dunnett Post-hoc test.

P values < 0.05 were considered to be statistically significant between the treatments.

## Results

### Characterisation of fermentation microbiota

**Figure 1.**
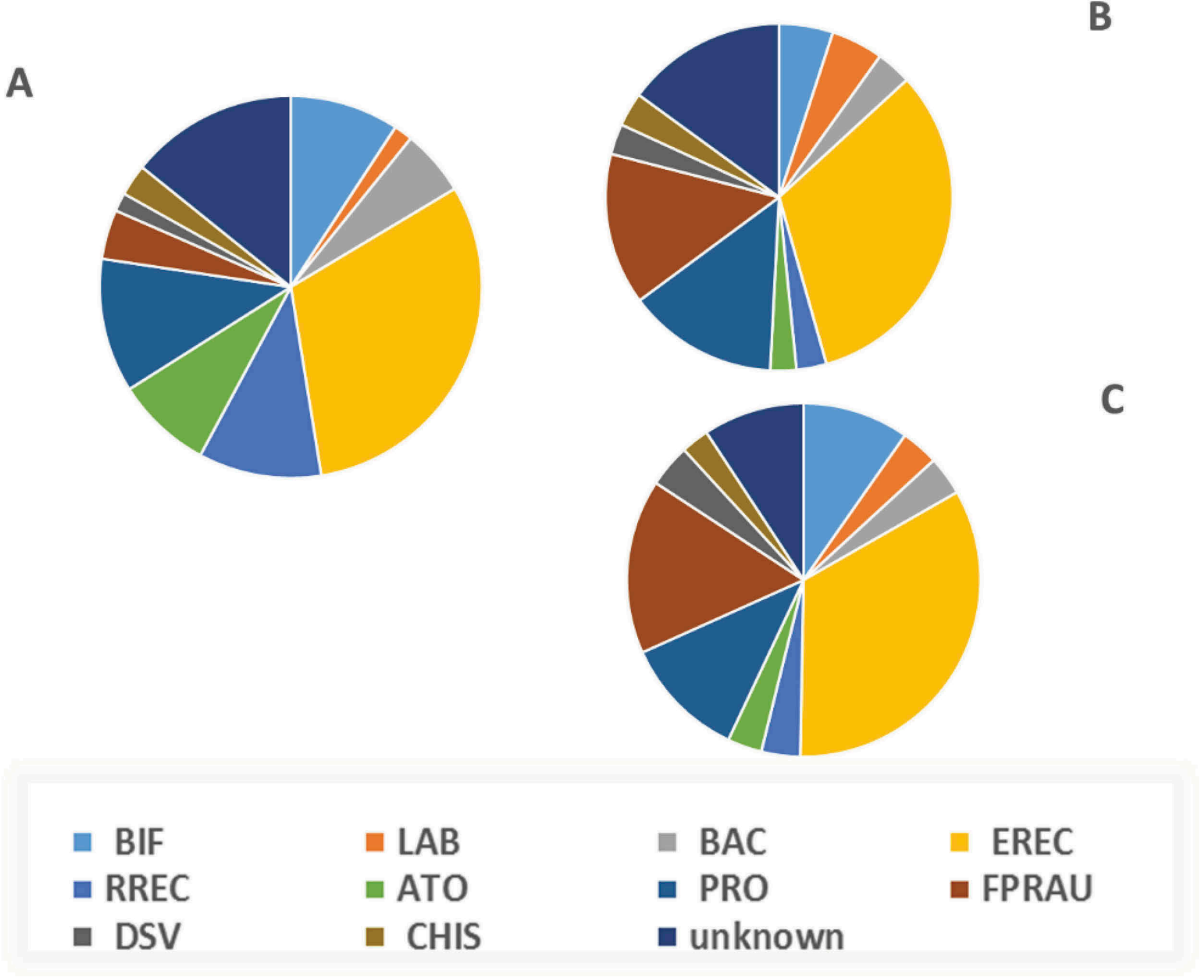
A, B and C: Microbial differences in starting inoculate composition used within *in vitro* batch culture fermentation from three donor samples. BIF- Bif164 positive count, LAB- Lab158 positive count, BAC- Bac 303 positive count, EREC- Erec 482 positive count, RREC- Rrec584 positive count, ATO- Ato 291 positive count, PRO- Prop853 positive count, FPRAU- Fprau647 positive count, DSV- Dsv687 positive count, CHIS- Chis150 positive count (see Table1).

Three faecal donors supplied specimens for the inter-individual biological replication of batch culture fermentations. An analysis of the microbial composition of the fermentation inoculate, was performed using 16S rRNA adherent molecular probes. There were statistically significant differences in the starting microbial composition of the batch culture fermentations which was reflected by subsequent inter-individual differences in metabolite production. At baseline, volunteer 1 had a greater relative abundance of strains staining positive for the *Bifidobacterium* (BIF164) P < 0.0001, *Atopobium* cluster (ATO 291) P < 0.0001, and *Desulfovibrio* (DSV 687) P < 0.0001 than volunteers 2 and 3. In contrast, volunteer 1 had a lower relative abundance of bacteria staining positive for *Faecalibacterium* (FPRAU 655) P < 0.000, *Propionibacterium* (Prop 853) P = 0.002, and *Lactobacillus* (Lab 158) P < 0.000 Figure 1.

Fermentation of the protein/amino acid and carbohydrate substrates resulted in changes in bacterial groups. Low tyrosine medium induced no significant bacteriological changes whilst high tyrosine medium led to an increase in total bacteria after 4h fermentation compared with baseline. The addition of FOS to low tyrosine medium resulted in significant increases in bifidobacteria at 4h and of total bacteria and *C. histolyticum* group at 8 h when compared to baseline. At 48h in the low tyrosine with FOS treatment there were more bacteroides, compared to the blank vessel **Supplementary data table 1**.

In the presence of FOS, the fermentation of high tyrosine resulted in significant increases in bacteria within the *E. rectales* group at t4; lactobacilli at t8 and *Roseburia* at t 24 as compared to t0. When compared to the blank vessel fermentation of high tyrosine with FOS resulted in more total bacteria, bacteria in the *Atopobioum* group and members of the *Faecalibacterium prausnitzii* group. **Supplementary data table 1**.

Supplementary albumin led to a reduction from baseline in the abundance of bacteria staining positive clostridia cluster IX at t4, increased *bacteroides* at t8 and increased *Atopobium* at t30. Compared to the blank vessel, albumin lead to enhanced lactobacilli at t30 and reduced DSV following 48 hours fermentation. Fermentation of soybean protein resulted in no significant changes of the groups monitored **Supplementary data table 1**

Fermentation of FOS resulted in increases in bacteroides after 4h, *bifidobacteria* and *bacteroides* after 30 h and total bacteria after 48 h as compared to t0. When compared to the blank vessel, FOS resulted in significant increases in *bifidobacteria* after 30 h, total bacteria after 8 h and *Atopobium* after 48 hours fermentation. Fermentation of peptone resulted in increases in *E. rectales* group after 8 h fermentation and total bacteria and *Roseburia* after 24 hours. **Supplementary data table 1**.

### Cresol, phenol and indole

**Figure 2.**
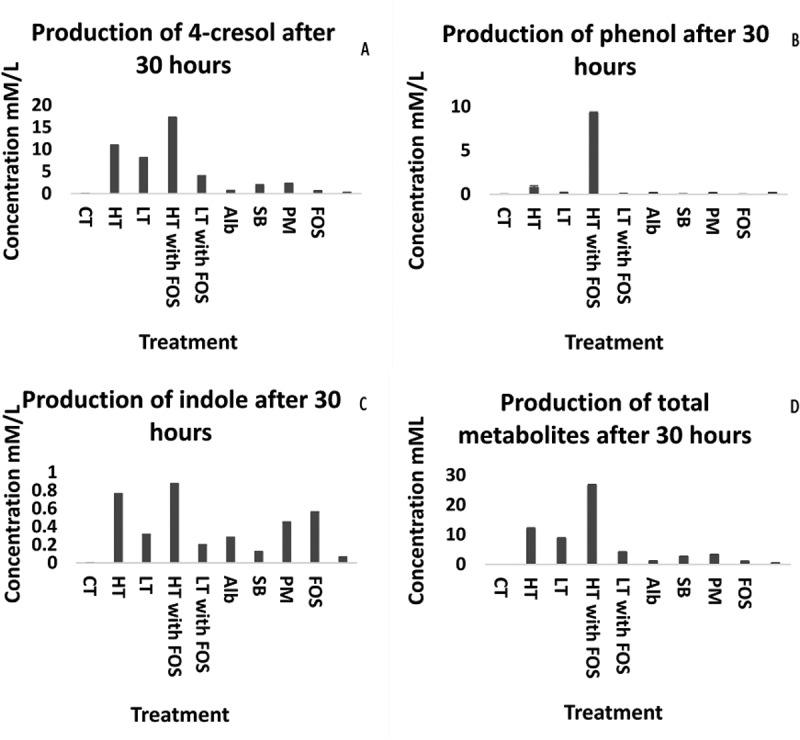
concentrations of p-cresol (A), phenol (B), indole (C) and total organic acid (D) using from mixed culture fermentation supernatants at 30 hours using faecal innoculate from volunteer 1; High tyrosine (HT) (0.3:100 w/w), Low tyrosine (LT) (0.003:100 w/w), High tyrosine with FOS (HT with FOS) (0.3:100 w/w and 1.5:100 w/w), Low tyrosine with FOS (LT with FOS) (0.003:100 w/w and 1.5:100 w/w), Soybean (SB) (0.3:100 w/w),, Peptone meat extract (PM) (0.3:100 w/w), and fructo-oligosaccharide (FOS) (1.5:100 w/w) after 30hrs incubation. The data presented as mean (± SEM) comparable to the control (n = 3).

There was considerable inter-individual variation in the synthesis of organic metabolites in the batch culture fermentation supernatants according to the donor inoculum used. For the purposes of clarity, Figure 2 shows metabolites produced with inoculate from volunteer 1 only, and shows variation in the production of proteolytic metabolites in fermentation supernatants at 30 hours, using different fermentation substrates (three biological replicates with the same faecal donor). (See supplementary Figure 1 for a comparison of inter-individual metabolite production). P-cresol was produced in the highest concentrations using a basal medium supplemented with a mixture of FOS (1.5% w/w) and tyrosine (0.3% w/w), reaching a concentration of 17.2 mM. Modest concentrations of p-cresol were also observed in batch cultures supplemented with tyrosine alone at high (0.3% w/w; reaching a concentration of 12 mM) or low doses (0.003% w/w). Lower concentrations of p-cresol were produced in fermentations where the media was supplemented with meat peptone (0.3% w/w), soy protein (0.3% w/w) or albumin (0.3% w/w). Using supplementary FOS alone in the media was not associated with appreciable p-cresol production (Figure 2A).

The highest concentrations of phenol (9.3 mM) were observed in the fermentation supernatant where the culture media was supplemented both tyrosine and FOS (0.3% w/w and 1.5 % w/w). With all other substrates phenol concentrations were much lower (Figure 2B). Indole production was also greatest (0.8 mM) in the fermentation supplemented with the combination of high tyrosine and FOS and lowest (0.06mM) in fermentations supplemented with FOS alone (Figure 2C).

### Short chain fatty acids

**Figure 3.**
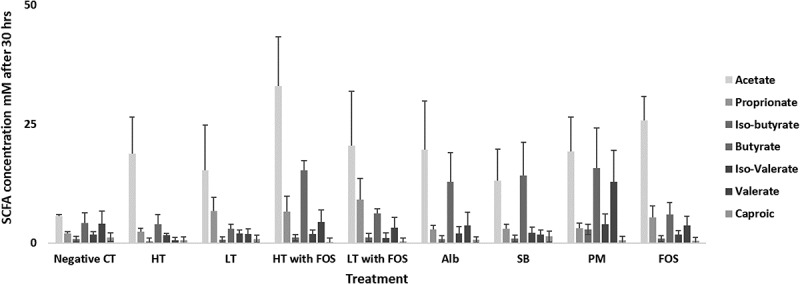
Concentration of SCFA in mixed culture fermentation supernatants at 30 hours using faecal inoculate from volunteer 1; High tyrosine (HT) (0.3:100 w/w), Low tyrosine (LT) (0.003:100 w/w), High tyrosine with FOS (HT with FOS) (0.3:100 w/w and 1.5:100 w/w), Low tyrosine with FOS (LT with FOS) (0.003:100 w/w and 1.5:100 w/w), Soybean (SB), Peptone meat extract (PM) and fructo-oligosaccharide (FOS) (1.5:100 w/w) after 30 hrs incubation. The data presented as mean (± SEM) comparable to the control (*n *= 3).

Inter-individual variation in the synthesis of SCFA in batch culture fermentation was observed according to the inoculum used; again for the purposes of clarity, data are presented here for volunteer 1 only (see supplementary **Figure 2** for comparisons with volunteers 2 and 3). Figure 3 shows the SCFA concentrations of fermentation supernatants at 30 h with different supplementary substrates (three biological replicates with the same faecal inoculum). The highest production of SCFA was observed using a basal media supplemented with a mixture of tyrosine (0.3% w/w) and FOS (1.5 w/w), reaching concentrations of 33 mM of acetate, 9 mM propionate and 6 mM butyrate. As anticipated, the presence of supplemental FOS led to higher SCFA concentrations with or without sources of supplemental nitrogen. The lowest concentrations of SCFA were produced in the negative control, indicating the baseline potential of the microbiota to produce SCFAs without the additional of substrates to the media.

### Genotoxicity of fermentation supernatants

**Figure 4.**
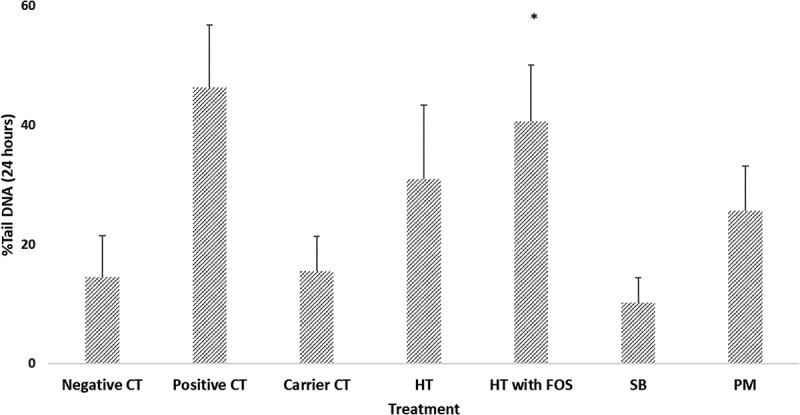
Effect of different fermentation supernatants (High tyrosine (HT), High tyrosine with FOS (HT with FOS), Soybean (SB) and Peptone meat (PM) on DNA damage for 24 hrs exposure on HT29 cell line. The data presented as mean (± SEM) percentage of DNA damage comparable to the control (*n *= 3). * indicate a significant difference compared to the untreated control (Dunnett test; *p < 0.05).

For clarity, in Figure 4 we present the induction of DNA damage in HT29 cells by fermentation supernatants by supplemental substrate for volunteer 1. DNA damage was assessed via the COMET assay following a 24 hour exposure to the filter sterilised supernatant at 10% of the carrier media. The highest observed levels of DNA damage were reported for the fermentation supernatant with the high tyrosine supplementation (with and without FOS), they were lowest in the fermentations supplemented with FOS alone. The low tyrosine, low tyrosine with FOS, albumin, soybean and peptone meat fermentations all produced moderately genotoxic fermentation samples.

Using the genotoxicity and metabolite data from all three volunteers we were able to regress the measured metabolites for individual fermentation supernatants against the reported genotoxicity. The best predictors of genotoxicity were p-cresol, acetate and iso-valerate. Of note however, acetate and iso-valerate were independently strong correlates of p-cresol (p = 0.001 and < 0.0001 respectively).

### P-cresol genotoxicity and cytotoxicity

**Figure 5.**
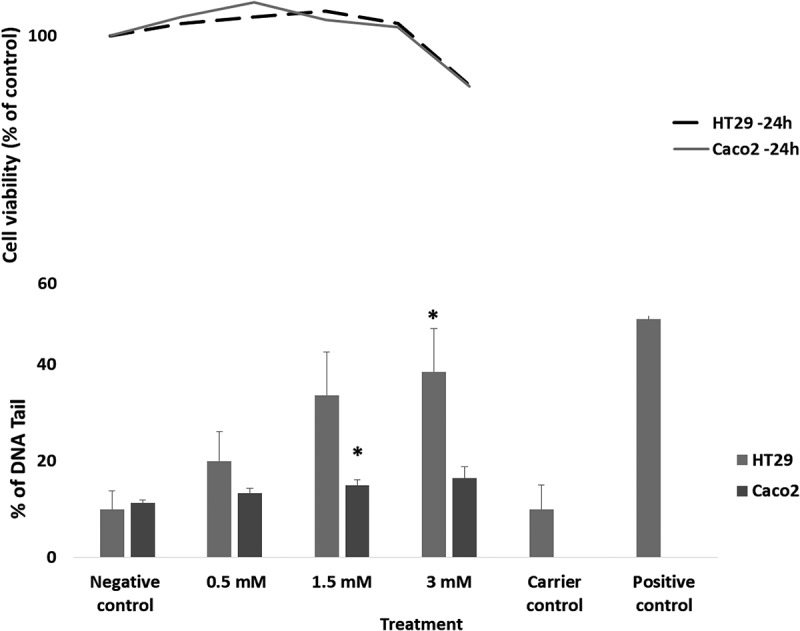
In the top plane we present the cytotoxicity of p-cresol (cell viability), mean percentage of viable cells comparable to the control (± SEM). Cells were incubated with increasing concentrations of p-cresol for 24 hours. The lower pane shows DNA strand breaks in HT29 and Caco-2 cells with increasing p-cresol concentrations: 0, 0.5, 1.5 and 3.0mM after 24 hours. Values are means ± SEM biological replicates. * indicate a significant difference compared to the untreated control (Dunnett test; *p < 0.05).

The genotoxic effects of increasing concentrations of p-cresol against both HT29 and Caco-2 cells, following a 24 h treatment, are shown in Figure 5. In the top pane of the figure we have plotted cell viability established via DAPI assay with equivalent exposures. Cell viability was maintained above 85 % at each of the doses used although there was a trend towards increasing cytotoxicity at 3 mM Figure 5.

There was a dose dependent increase in DNA damage with increasing concentrations of 4- cresol reaching statistical significance at concentrations of 3 mM for both cells lines (p = < 0.05) the observed DNA damage was higher in the HT29 cells than in the Caco-2 cell line.

**Figure 6.**
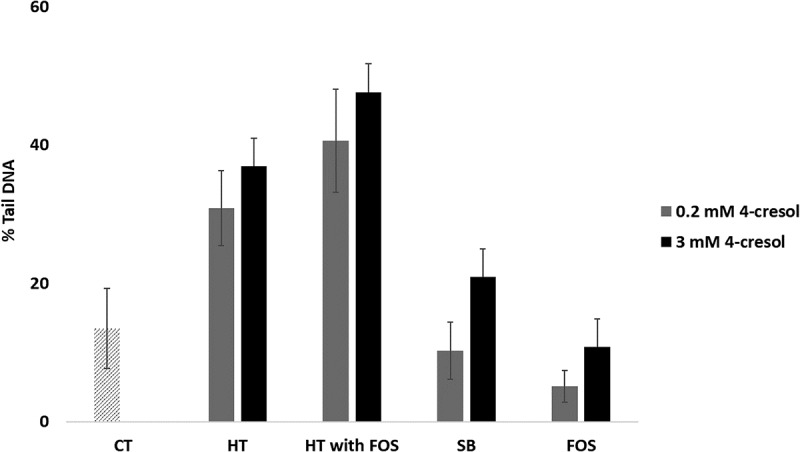
Comet data from fermentation supernatants spiked with a low dose (0.2mM), or a high dose (3mM) of p-cresol. The spiked supernatants were of High tyrosine (HT), High tyrosine with FOS (HT with FOS), and from soybean (SB) and FOS alone. DNA damage was assessed after 24 hr incubation in the HT29 cell line. The data presented as mean (± SEM) percentage of DNA damage compared to the control (n = 3). (Dunnett test; *p < 0.05).

Internally spiked fermentation supernatants: To consider the effects of increasing p-cresol concentrations within the context of the gut microbial environment we spiked selected fermentation supernatants, post-fermentation with either a low (0.2mM) or high (3mM) doses of p-cresol and assessed genotoxicity against HT29 cells Figure 6. With higher concentrations of exogenous 4 cresol we observed an increase in DNA strand breaks.

### P-cresol and cell cycle kinetics

**Figure 7.**
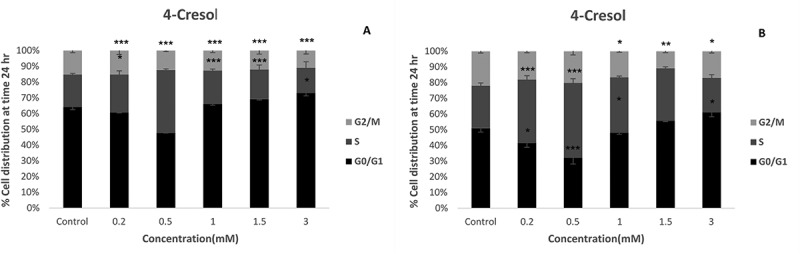
Cell cycle progression of HT29 (A) and Caco-2 (B) cell line treated with 0.2, 0.5, 1.0, 1.5 and 3mM of p-cresol for 24hours. The percentage of cells in each phase of the cell cycle was determined using flow cytometry to quantify DNA content (n = 4). Asterisks indicate a significant difference compare to the untreated control (Dunnett test; * p < 0.05, ** p < 0.01, ***p < 0.001).

We treated both the HT29 (A) and Caco-2 (B) cells with 4 cresol for 24 hours, before observing disruptions to cell cycle behaviour Figure 7. There was a non-linear dose response to p-cresol, with the changes observed perhaps relating to levels of DNA damage. At lower exposures of up to 0.5 mM p-cresol, we observed decreases in the abundance of cells in *G_0/_G_1_* with a compensatory increase in the proportion of cells in *S* phase, however at higher concentrations the proportion of cells in *G_0/_G_1_* increased significantly relative to the proportion of cells in *S* phase in both cell lines, suggesting a slight growth promoting effect at lower doses and *G_0/_G_1_* growth arrest in response to genotoxic insult at higher doses.

## Discussion

In a gut fermentation model, we first observed that the source of the faecal inoculum influenced the subsequent metabolic profile of gut model supernatants given the same supplementary substrate; at baseline, the faecal inoculate from volunteer 1 was characterised by a higher relative proportion of *Bifidobacterium* (BIF 164), *Atopobium* cluster (ATO 291) and *Desulfovibrio* (DSV 687) than volunteers 2 and 3; and a lower relative abundance of bacteria staining positive for *Faecalibacterium* (FPRAU 655) *Propionibacterium* (Prop 853) and *Lactobacillus* (Lab 158) . The microbial composition of the faecal inoculate from volunteers 2 and 3 were more closely aligned with each other and were associated with a lower total production of p-cresol and other metabolites than the fermentation supernatants from volunteer 1. A heterogenic metabolite response between individuals consuming the same foods according to their microbial composition is well established, but here our observation provided a rationale for focussing on metabolite responses in repeat fermentations using inoculate from one volunteer in our subsequent analysis of fermentation metabolite interactions with epithelial cells.

We then demonstrated that both the microbial composition and the metabolite profiles of the fermentation supernatants were influenced by the supplemental fermentation substrates. For example, the total bacteria count was significantly increased following fermentation with medium supplemented with high levels of tyrosine, high tyrosine plus FOS, or with peptone meat extract. **(Supplementary Table 1)**. With the addition of FOS together with tyrosine, a large increase in total bacteria was observed, coupled to compositional changes in the abundance of *Roseburia*, a key butyrate producing group, but also in *Atopobium* and *Faecalibacterium prausnitzii*. Of the supplemental protein sources, only albumin statistically significantly increased the relative abundance of microbes staining for *Lactobacillus* at 30 hours and *Desulfovibrio* at 48 hours compared to control. These shifts in composition might be considered relatively minor, and this may reflect the influence of a relatively high protein western habitual diet on the donors faecal inoculum; we know from observational studies, that higher animal protein diets are associated with lower microbial diversity, reductions in the abundance of saccharolytic bacteria and increases in potentially harmful species such as *Fusobacterium nucleatum, Rhodopseudomonas faecalis, Bacteroides vulgatus and Enterococcus faecalis*.,^17,18^

In contrast to the minor changes in microbial composition, we observed quite notable changes in microbial activity as reflected in our metabolite profiles. Supplementing broths with tyrosine, or with sources of protein, induced increases in the production of the proteolytic metabolites, but also increased the production of acetate and butyrate. This observation is consistent with the findings of a recent 3-week intervention study in volunteers given high protein diets; the volunteers microbial composition did not change, but there was a transition towards more proteolytic metabolite production.^19^ As expected, supplementing fermentation broth with the prebiotic FOS led to enhanced synthesis of the saccharolytic metabolites (SCFA) with little effect on the production of cresol, indole or phenol. Furthermore, combining prebiotic FOS with supplemental tyrosine in the fermentation broth led to the highest overall concentrations of proteolytic end products in any of our fermentation supernatants. We had anticipated that the FOS might suppress the activity of proteolytic species, in practice the FOS increased the overall abundance of bacteria, relative to fermentations with tyrosine alone, and this increased biomass might explain the greater production of proteolytic products. The SCFA are generally seen as beneficial products of both proteolytic and saccharolytic fermentation; whereas indoles, phenols and cresol might be viewed as potential toxins produced during proteolytic fermentation. From a physiological perspective, the enhanced SCFA produced when FOS was present with tyrosine might counter some of the potential harm associated with the proteolytic metabolites.^20^

We next evaluated the fermentation supernatants for their capacity to induce DNA damage in human intestinal cell lines. The substrate influenced the level of DNA damage observed, with the high tyrosine plus FOS fermentation supernatant being the most genotoxic. We used a regression model to evaluate the relationships between the observed metabolites in the supernatant and genotoxicity. From this analysis p-cresol emerged as the strongest predictor of DNA damage (Table 2).

**Table 2.** Linear regression model; metabolite predictors of supernatant genotoxicity.

	Metabolite predictor	r	*P value*
Model 1	p-cresol	0.775	0.002
	Phenol		0.317
	indole		0.708
Model 2	p-cresol	0.681	0.005
Model 3	SCFA		
	Acetate	0.759	0.001
	Iso-valerate	0.995	0.000

p-Cresol can be found in human faeces at concentrations of up to 0.5 mM; *in vivo* p-cresol is largely absorbed and metabolised to p-cresol sulphate appearing in urine at concentrations of up to 0.3 mM.^21^ Therefore faecal p-cresol probably under-represents colonic concentrations. In our *in vitro* batch culture fermentation system, p-cresol concentrations reached up to 17mM, however this gut model has no mechanism to remove accumulating metabolites that are otherwise absorbed in the intestinal tract. Probable physiological intestinal epithelial exposures are likely in the low mM range as reported *in situ* by Smith and Macfarlane.^22^

When applied to two different colorectal cultured cell lines at these physiologically relevant concentrations p-cresol proved cytotoxic at doses of over 3 mM. Genotoxicity was therefore assessed at doses of up to 3 mM. We observed a linear dose dependent increase in DNA damage in both cell lines but the HT29 cells appeared more sensitive to p-cresol mediated genotoxicity than the Caco-2 cells. Importantly, we then applied p-cresol to the cell lines as a spiked component of the gut model fermentation supernatants, and thus demonstrated that p-cresol may contribute to genotoxicity as part of the gut fermentation milieu. Our observations are consistent with previous work by Andriamihaja et al, who used the γH2AX assay and also observed genotoxicity in both HT29 Glc^−/+^ and LS-174T human colonic cell lines at concentrations of > 1.5 mM.^23^

We then studied the effects of p-cresol on cell cycle activity. Again consistent with the observations of Andriamihaja et al^23^, at low concentrations we observed that the abundance of cells in *S* phase was increased in both cell lines with a subsequent decrease in the abundance of cells in *G0/G1*. Assuming this is a mitogenic response, and not an activation of *S* phase arrest, it could explain previously reported tumour promotion by p-cresol in a classical murine papilloma study following tumour initiation with 9,10-dimethyl-l-benzanthracene.^24^ At higher doses we observed a reduction in the proportion of cells in *S* phase and an increase in proportion of cells in *G_0_/G_1_* and in *G_2_/M*, perhaps indicating cell cycle arrest in response to DNA damage.

The models we have employed in this study are widely used and accepted in mechanistic studies of dietary exposures related to colorectal cancer; they represent different aspects of the carcinogenic process. Having said that, these are *in vitro* systems, the anti-cancer defence mechanisms of the colonic epithelium are potentially very different *in vivo*, and the complexity of the microbiota and the environment of the gut lumen confounds our ability to draw firm conclusions regarding the potential carcinogenic effects of p-cresol *in vivo*.^25^ To date, one human randomised crossover trial with high and low protein diets has reported a weak correlation between urinary p-cresol excretion and FW genotoxicity^26^, whilst the human dietary intervention study by Beaumont et al. reported changes in the expressions of genes involved in cell death and cell kinetics in volunteers consuming higher protein diets, who also showed increased p-cresol excretion.^19^ Based on a very small (n = 6 cases) case control study, Bone and Tamm^27^ argued that comparable urinary concentrations of p-cresol from volunteers with bowel cancer to healthy controls was evidence that this metabolite is not affecting CRC risk; ours and other emerging data might challenge this. Evaluation of p-cresol in stored urinary samples from existing prospective cohort studies may help establish the strength of any relationship with cancer risk and potentially validate the use of urinary p-cresol as a biomarker of risk for use in intervention studies.
