# Supplementary material for: Modelling the role of microbial p-cresol in colorectal genotoxicity
Source: Gut Microbes. 2018 Oct 25;10(3):398–411. doi: 10.1080/19490976.2018.1534514 (PMC6546321; doi:10.1080/19490976.2018.1534514)
Supplement: Supplemental Material [file kgmi-10-03-1534514-s001.docx]

**Supplementary data**

**Supplementary table 1:** Microbial counts following fermentation of substrates in pH controlled batch cultures. Log_10_ number of bacterial cells/ml batch supernatant, +/- stdev. * indicates significantly different to blank, ^a^ indicates significantly different to t0.

| **TYR (L)** | **t0** |  |  | **t4** |  |  | **t8** |  |  | **t24** |  |  | **t30** |  |  | **t48** |  |  |
| --- | --- | --- | --- | --- | --- | --- | --- | --- | --- | --- | --- | --- | --- | --- | --- | --- | --- | --- |
| EUB FL2 | **6.71** | ±0.74 |  | **7.03** | ±0.75 |  | **6.92** | ±0.61 |  | **6.96** | ±0.74 |  | **6.92** | ±0.18 |  | **6.74** | ±0.59 |  |
| BIF | **5.68** | ±0.50 |  | **5.91** | ±0.74 |  | **5.79** | ±0.74 |  | **5.93** | ±0.70 |  | **5.76** | ±0.38 |  | **5.63** | ±0.65 |  |
| LAB | **5.15** | ±0.98 |  | **5.69** | ±0.73 |  | **5.33** | ±0.67 |  | **5.64** | ±0.71 |  | **5.67** | ±0.36 |  | **5.60** | ±0.79 |  |
| BAC | **5.30** | ±0.71 |  | **5.69** | ±0.72 |  | **5.66** | ±0.57 |  | **5.79** | ±0.72 |  | **5.78** | ±0.23 |  | **5.78** | ±0.65 |  |
| EREC | **6.19** | ±0.73 |  | **6.51** | ±0.77 |  | **6.32** | ±0.65 |  | **6.15** | ±0.84 |  | **5.88** | ±0.43 |  | **5.81** | ±0.55 |  |
| RREC | **5.36** | ±0.72 |  | **5.29** | ±0.95 |  | **5.55** | ±0.46 |  | **5.44** | ±0.83 |  | **5.62** | ±0.22 |  | **5.55** | ±0.51 |  |
| ATO | **5.41** | ±0.43 |  | **5.69** | ±0.37 |  | **5.53** | ±0.39 |  | **5.88** | ±0.53 |  | **5.72** | ±0.40 |  | **5.70** | ±0.46 |  |
| PRO | **5.82** | ±0.83 |  | **5.88** | ±0.92 |  | **6.05** | ±0.68 |  | **6.03** | ±0.94 |  | **6.17** | ±0.31 |  | **5.88** | ±0.67 |  |
| FPRAU | **5.69** | ±0.88 |  | **6.06** | ±1.14 |  | **5.89** | ±0.91 |  | **5.95** | ±0.78 |  | **5.79** | ±0.41 |  | **5.69** | ±0.54 |  |
| DSV | **5.11** | ±0.90 |  | **5.48** | ±0.82 |  | **5.39** | ±0.55 |  | **5.55** | ±0.62 |  | **5.54** | ±0.38 |  | **5.26** | ±0.68 |  |
| CHIS | **5.27** | ±0.77 |  | **5.55** | ±0.56 |  | **5.29** | ±0.65 |  | **5.64** | ±0.48 |  | **5.61** | ±0.33 |  | **5.50** | ±0.61 |  |

| **TYR (H)** | **t0** |  |  | **t4** |  |  | **t8** |  |  | **t24** |  |  | **t30** |  |  | **t48** |  |  |
| --- | --- | --- | --- | --- | --- | --- | --- | --- | --- | --- | --- | --- | --- | --- | --- | --- | --- | --- |
| EUB FL1 | **6.99** | ±0.70 |  | **7.19** | ±0.69 | a | **7.13** | ±0.46 |  | **7.07** | ±0.81 |  | **7.16** | ±0.20 |  | **6.85** | ±1.00 |  |
| BIF | **5.87** | ±0.61 |  | **5.85** | ±0.79 |  | **5.94** | ±0.47 |  | **6.04** | ±0.71 |  | **5.82** | ±0.34 |  | **5.78** | ±0.90 |  |
| LAB | **5.46** | ±0.95 |  | **6.01** | ±1.10 |  | **5.96** | ±0.88 |  | **5.79** | ±1.22 |  | **6.10** | ±0.62 |  | **5.61** | ±1.22 |  |
| BAC | **5.59** | ±0.57 |  | **5.87** | ±0.70 |  | **5.76** | ±0.34 |  | **5.86** | ±0.67 |  | **5.87** | ±0.22 |  | **5.74** | ±0.85 |  |
| EREC | **6.50** | ±0.71 |  | **6.64** | ±0.62 |  | **6.46** | ±0.53 |  | **6.31** | ±1.01 |  | **6.11** | ±0.14 |  | **5.89** | ±0.95 |  |
| RREC | **5.66** | ±0.39 |  | **5.52** | ±0.36 |  | **5.67** | ±0.34 |  | **5.69** | ±0.74 |  | **5.75** | ±0.14 |  | **5.48** | ±1.08 |  |
| ATO | **5.59** | ±0.42 |  | **5.69** | ±0.58 |  | **5.69** | ±0.16 |  | **5.96** | ±0.51 |  | **6.27** | ±0.41 |  | **5.80** | ±0.71 |  |
| PRO | **6.07** | ±0.73 |  | **6.06** | ±0.95 |  | **6.31** | ±0.58 |  | **6.16** | ±1.03 |  | **6.18** | ±0.63 |  | **6.11** | ±1.23 |  |
| FPRAU | **5.98** | ±1.01 |  | **6.13** | ±0.96 |  | **6.10** | ±0.67 |  | **5.94** | ±0.77 |  | **5.89** | ±0.02 |  | **5.63** | ±0.94 |  |
| DSV | **5.40** | ±0.87 |  | **5.51** | ±0.58 |  | **5.50** | ±0.40 |  | **5.56** | ±0.78 |  | **5.67** | ±0.22 |  | **5.31** | ±0.99 |  |
| CHIS | **5.43** | ±0.72 |  | **5.58** | ±0.43 |  | **5.55** | ±0.41 |  | **5.68** | ±0.69 |  | **5.76** | ±0.31 |  | **5.42** | ±0.95 |  |

| **TYR (L)+FOS** | **t0** |  |  | **t4** |  |  | **t8** |  |  | **t24** |  |  | **t30** |  |  | **t48** |  |  |
| --- | --- | --- | --- | --- | --- | --- | --- | --- | --- | --- | --- | --- | --- | --- | --- | --- | --- | --- |
| EUB FL4 | **7.00** | ±0.81 |  | **7.33** | ±0.78 |  | **7.44** | ±0.86 | a | **6.59** | ±1.37 |  | **7.17** | ±1.47 |  | **7.57** | ±0.13 |  |
| BIF | **5.78** | ±0.96 |  | **6.28** | ±0.94 | a | **6.52** | ±1.22 |  | **5.75** | ±1.62 |  | **6.26** | ±1.51 |  | **6.45** | ±0.83 |  |
| LAB | **5.53** | ±0.91 |  | **6.22** | ±0.89 |  | **6.22** | ±0.92 |  | **4.99** | ±1.18 |  | **5.71** | ±1.53 |  | **6.31** | ±0.43 |  |
| BAC | **5.33** | ±1.21 |  | **5.93** | ±0.86 |  | **6.20** | ±1.15 |  | **5.62** | ±1.45 |  | **6.26** | ±1.64 |  | **6.52** | ±0.22 | * |
| EREC | **6.38** | ±0.98 |  | **6.62** | ±0.82 |  | **6.48** | ±0.77 |  | **5.55** | ±1.38 |  | **6.00** | ±1.22 |  | **6.21** | ±0.17 |  |
| RREC | **5.34** | ±1.10 |  | **5.75** | ±0.46 |  | **5.85** | ±0.56 |  | **5.17** | ±1.11 |  | **5.69** | ±1.20 |  | **5.98** | ±0.19 |  |
| ATO | **5.55** | ±0.64 |  | **5.82** | ±0.16 |  | **6.12** | ±0.65 |  | **5.56** | ±1.67 |  | **5.98** | ±1.76 |  | **6.38** | ±0.44 |  |
| PRO | **6.28** | ±0.44 |  | **6.17** | ±1.04 |  | **6.15** | ±1.09 |  | **5.52** | ±1.45 |  | **6.05** | ±2.03 |  | **6.80** | ±0.57 |  |
| FPRAU | **5.99** | ±1.31 |  | **6.34** | ±0.91 |  | **6.32** | ±0.93 |  | **5.47** | ±0.98 |  | **6.03** | ±1.16 |  | **6.18** | ±0.12 |  |
| DSV | **5.29** | ±1.18 |  | **5.69** | ±0.88 |  | **5.59** | ±0.94 |  | **4.97** | ±1.21 |  | **5.57** | ±1.75 |  | **5.91** | ±0.18 |  |
| CHIS | **5.31** | ±1.16 |  | **5.89** | ±1.00 |  | **6.16** | ±1.05 | a | **5.14** | ±1.02 |  | **5.83** | ±1.14 |  | **6.15** | ±0.16 |  |

| **TYR (H)+FOS** | **t0** |  |  | **t4** |  |  | **t8** |  |  | **t24** |  |  | **t30** |  |  | **t48** |  |  |
| --- | --- | --- | --- | --- | --- | --- | --- | --- | --- | --- | --- | --- | --- | --- | --- | --- | --- | --- |
| EUB FL3 | **6.98** | ±0.83 |  | **7.42** | ±0.98 |  | **7.33** | ±1.01 |  | **7.31** | ±0.54 |  | **7.93** | ±0.32 | * | **7.59** | ±0.30 |  |
| BIF | **5.89** | ±0.68 |  | **6.45** | ±1.06 |  | **6.42** | ±1.26 |  | **6.53** | ±0.84 |  | **6.84** | ±0.77 |  | **6.58** | ±0.75 |  |
| LAB | **5.45** | ±0.97 |  | **6.30** | ±1.31 |  | **5.97** | ±1.06 | a | **5.93** | ±0.85 |  | **6.62** | ±0.79 |  | **6.30** | ±1.04 |  |
| BAC | **5.54** | ±0.73 |  | **6.07** | ±1.04 |  | **6.16** | ±0.89 |  | **5.64** | ±0.18 |  | **6.58** | ±0.73 |  | **6.24** | ±0.21 |  |
| EREC | **6.41** | ±0.90 |  | **6.68** | ±0.89 | a | **6.55** | ±1.14 |  | **6.28** | ±0.81 |  | **6.70** | ±0.04 |  | **6.30** | ±0.53 |  |
| RREC | **5.65** | ±0.63 |  | **5.74** | ±0.60 |  | **5.44** | ±0.66 |  | **5.41** | ±0.57 | a | **6.08** | ±0.49 |  | **5.88** | ±0.37 |  |
| ATO | **5.66** | ±0.48 |  | **5.97** | ±0.74 |  | **5.69** | ±0.77 |  | **5.96** | ±0.72 |  | **6.60** | ±0.45 | * | **6.34** | ±0.43 |  |
| PRO | **6.05** | ±0.87 |  | **6.17** | ±1.12 |  | **6.42** | ±0.75 |  | **6.16** | ±0.63 |  | **6.73** | ±1.09 |  | **6.61** | ±0.87 |  |
| FPRAU | **6.09** | ±1.05 |  | **6.37** | ±1.04 |  | **6.28** | ±1.24 |  | **6.18** | ±0.50 |  | **6.60** | ±0.18 | * | **6.11** | ±0.29 |  |
| DSV | **5.56** | ±0.93 |  | **5.84** | ±0.87 |  | **5.56** | ±0.78 |  | **5.35** | ±0.32 |  | **6.14** | ±0.44 |  | **5.71** | ±0.56 |  |
| CHIS | **5.56** | ±0.79 |  | **5.84** | ±0.91 |  | **5.83** | ±1.08 |  | **5.49** | ±0.23 |  | **6.21** | ±0.62 |  | **5.92** | ±0.50 |  |

| **ALBUMIN** | **t0** |  |  | **t4** |  |  | **t8** |  |  | **t24** |  |  | **t30** |  |  | **t48** |  |  |
| --- | --- | --- | --- | --- | --- | --- | --- | --- | --- | --- | --- | --- | --- | --- | --- | --- | --- | --- |
| EUB FL5 | **7.07** | ±0.44 |  | **7.00** | ±0.47 |  | **7.13** | ±0.52 |  | **7.02** | ±0.36 |  | **7.09** | ±0.05 |  | **6.73** | ±0.28 |  |
| BIF | **5.90** | ±0.47 |  | **5.79** | ±0.59 |  | **5.94** | ±0.73 |  | **6.02** | ±0.35 |  | **5.90** | ±0.37 |  | **5.68** | ±0.47 |  |
| LAB | **5.57** | ±0.73 |  | **5.71** | ±0.13 |  | **5.49** | ±0.58 |  | **5.69** | ±0.43 |  | **5.85** | ±0.05 | * | **5.59** | ±0.23 |  |
| BAC | **5.75** | ±0.36 |  | **5.70** | ±0.38 |  | **6.03** | ±0.30 | a | **5.75** | ±0.09 |  | **5.84** | ±0.26 |  | **5.70** | ±0.28 |  |
| EREC | **6.56** | ±0.39 |  | **6.44** | ±0.53 |  | **6.46** | ±0.67 |  | **6.02** | ±0.25 |  | **6.11** | ±0.15 |  | **5.62** | ±0.48 |  |
| RREC | **5.63** | ±0.47 |  | **5.36** | ±0.54 |  | **5.68** | ±0.50 |  | **5.64** | ±0.29 |  | **5.51** | ±0.33 |  | **5.56** | ±0.23 |  |
| ATO | **5.72** | ±0.26 |  | **5.63** | ±0.71 |  | **5.67** | ±0.37 |  | **6.04** | ±0.60 |  | **6.09** | ±0.23 | a | **5.60** | ±0.41 |  |
| PRO | **6.13** | ±0.59 |  | **5.99** | ±0.61 | a | **6.31** | ±0.52 |  | **6.03** | ±0.64 |  | **6.28** | ±0.46 |  | **5.87** | ±0.43 |  |
| FPRAU | **6.12** | ±0.69 |  | **6.03** | ±0.78 |  | **6.12** | ±0.89 |  | **6.03** | ±0.51 |  | **5.97** | ±0.20 |  | **5.62** | ±0.46 |  |
| DSV | **5.54** | ±0.65 |  | **5.33** | ±0.52 |  | **5.40** | ±0.63 |  | **5.60** | ±0.17 |  | **5.66** | ±0.37 |  | **5.20** | ±0.29 | * |
| CHIS | **5.60** | ±0.52 |  | **5.22** | ±0.61 |  | **5.53** | ±0.59 |  | **5.56** | ±0.27 |  | **5.57** | ±0.10 |  | **5.49** | ±0.21 |  |

| **SOYBEAN** | **t0** |  |  | **t4** |  |  | **t8** |  |  | **t24** |  |  | **t30** |  |  | **t48** |  |  |
| --- | --- | --- | --- | --- | --- | --- | --- | --- | --- | --- | --- | --- | --- | --- | --- | --- | --- | --- |
| EUB FL6 | **7.10** | ±0.60 |  | **7.12** | ±0.41 |  | **7.13** | ±0.66 |  | **6.88** | ±0.87 |  | **6.84** | ±0.80 |  | **6.97** | ±0.67 |  |
| BIF | **5.94** | ±0.62 |  | **5.99** | ±0.63 |  | **6.00** | ±0.55 |  | **5.94** | ±0.63 |  | **5.84** | ±0.68 |  | **5.96** | ±0.73 |  |
| LAB | **5.68** | ±0.49 |  | **6.18** | ±0.44 |  | **5.96** | ±1.29 |  | **5.76** | ±1.34 |  | **5.90** | ±1.16 |  | **5.96** | ±0.97 |  |
| BAC | **5.58** | ±0.53 |  | **5.87** | ±0.36 |  | **5.83** | ±0.69 |  | **5.64** | ±0.94 |  | **5.58** | ±0.89 |  | **5.84** | ±0.88 |  |
| EREC | **6.52** | ±0.71 |  | **6.52** | ±0.44 |  | **6.31** | ±0.73 |  | **5.83** | ±0.97 |  | **5.78** | ±0.95 |  | **5.97** | ±0.66 |  |
| RREC | **5.64** | ±0.31 |  | **5.63** | ±0.57 |  | **5.70** | ±0.32 |  | **5.58** | ±0.86 |  | **5.59** | ±0.72 |  | **5.78** | ±0.61 |  |
| ATO | **5.59** | ±0.29 |  | **5.59** | ±0.25 |  | **5.59** | ±0.19 |  | **5.65** | ±0.69 |  | **5.61** | ±0.80 |  | **5.67** | ±0.77 |  |
| PRO | **6.29** | ±0.73 |  | **5.97** | ±0.52 |  | **6.21** | ±0.88 |  | **5.99** | ±1.12 |  | **5.58** | ±0.79 |  | **6.04** | ±0.50 |  |
| FPRAU | **6.19** | ±0.83 |  | **6.08** | ±0.66 |  | **5.98** | ±0.74 |  | **5.56** | ±0.84 |  | **5.57** | ±0.93 |  | **5.65** | ±0.72 |  |
| DSV | **5.59** | ±0.58 |  | **5.46** | ±0.46 |  | **5.59** | ±0.44 |  | **5.37** | ±0.98 |  | **5.41** | ±0.86 |  | **5.49** | ±1.05 |  |
| CHIS | **5.53** | ±0.58 |  | **5.46** | ±0.63 |  | **5.57** | ±0.44 |  | **5.44** | ±0.97 |  | **5.68** | ±0.75 |  | **5.65** | ±0.71 |  |

| **FOS** | **t0** |  |  | **t4** |  |  | **t8** |  |  | **t24** |  |  | **t30** |  |  | **t48** |  |  |
| --- | --- | --- | --- | --- | --- | --- | --- | --- | --- | --- | --- | --- | --- | --- | --- | --- | --- | --- |
| EUB FL6 | **6.81** | ±0.57 |  | **7.39** | ±0.60 |  | **7.82** | ±0.15 | * | **7.36** | ±0.80 |  | **7.71** | ±0.16 | * | **7.83** | ±0.19 | *a |
| BIF | **5.62** | ±0.60 |  | **6.21** | ±1.03 |  | **6.61** | ±1.12 |  | **6.42** | ±1.07 |  | **7.37** | ±0.17 | *a | **6.73** | ±0.79 |  |
| LAB | **5.41** | ±0.69 |  | **6.00** | ±1.02 |  | **6.49** | ±0.88 |  | **6.05** | ±1.42 |  | **6.68** | ±0.93 |  | **6.61** | ±0.98 |  |
| BAC | **5.43** | ±0.57 |  | **6.21** | ±0.56 | a | **6.36** | ±0.38 |  | **5.86** | ±0.86 |  | **6.32** | ±0.38 | a | **6.56** | ±0.70 |  |
| EREC | **6.32** | ±0.56 |  | **6.29** | ±0.40 |  | **6.49** | ±0.05 |  | **6.34** | ±0.33 |  | **6.36** | ±0.15 |  | **6.30** | ±0.40 |  |
| RREC | **5.44** | ±0.62 |  | **5.59** | ±0.36 |  | **5.76** | ±0.48 |  | **5.68** | ±0.57 |  | **5.97** | ±0.31 |  | **6.03** | ±0.73 |  |
| ATO | **5.42** | ±0.45 |  | **5.92** | ±0.78 |  | **6.02** | ±1.02 |  | **6.28** | ±0.87 |  | **6.22** | ±0.50 |  | **6.54** | ±0.26 | * |
| PRO | **5.79** | ±0.71 |  | **6.40** | ±0.78 |  | **6.83** | ±0.84 |  | **6.05** | ±0.81 |  | **6.17** | ±0.30 |  | **6.72** | ±1.14 |  |
| FPRAU | **5.89** | ±0.65 |  | **5.93** | ±0.62 |  | **6.18** | ±0.18 |  | **5.91** | ±0.60 |  | **6.20** | ±0.21 |  | **6.11** | ±0.49 |  |
| DSV | **5.40** | ±0.68 |  | **5.38** | ±0.32 |  | **5.61** | ±0.59 |  | **5.60** | ±0.69 |  | **5.83** | ±0.15 |  | **5.82** | ±0.80 |  |
| CHIS | **5.34** | ±0.60 |  | **5.99** | ±1.13 |  | **6.31** | ±0.23 | * | **5.74** | ±0.81 |  | **5.91** | ±0.26 |  | **5.94** | ±0.71 |  |

| **PEPTONE MEAT** | **t0** |  |  | **t4** |  |  | **t8** |  |  | **t24** |  |  | **t30** |  |  | **t48** |  |  |
| --- | --- | --- | --- | --- | --- | --- | --- | --- | --- | --- | --- | --- | --- | --- | --- | --- | --- | --- |
| EUB FL6 | **6.71** | ±0.66 |  | **6.89** | ±0.43 |  | **7.20** | ±0.47 |  | **7.01** | ±0.55 | a | **7.08** | ±0.13 |  | **7.00** | ±0.53 |  |
| BIF | **5.45** | ±0.70 |  | **5.74** | ±0.50 |  | **5.99** | ±0.54 |  | **5.97** | ±0.40 |  | **5.97** | ±0.32 |  | **5.91** | ±0.45 |  |
| LAB | **5.37** | ±0.66 |  | **5.24** | ±0.26 |  | **5.59** | ±0.82 |  | **5.76** | ±0.98 |  | **5.58** | ±0.26 |  | **5.76** | ±0.57 |  |
| BAC | **5.36** | ±0.60 |  | **5.63** | ±0.39 |  | **5.74** | ±0.97 |  | **5.82** | ±0.26 |  | **5.95** | ±0.34 |  | **6.11** | ±0.52 |  |
| EREC | **6.13** | ±0.64 |  | **6.34** | ±0.51 |  | **6.52** | ±0.48 | a | **6.34** | ±0.46 |  | **6.09** | ±0.15 |  | **6.10** | ±0.50 |  |
| RREC | **5.38** | ±0.53 |  | **5.34** | ±0.33 |  | **5.83** | ±0.44 |  | **5.58** | ±0.52 | a | **5.76** | ±0.29 |  | **5.72** | ±0.52 |  |
| ATO | **5.43** | ±0.38 |  | **5.47** | ±0.54 |  | **5.58** | ±0.38 |  | **5.77** | ±0.55 |  | **5.90** | ±0.08 |  | **5.80** | ±0.41 |  |
| PRO | **5.91** | ±0.70 |  | **6.00** | ±0.59 |  | **6.30** | ±1.12 |  | **5.99** | ±0.69 |  | **6.18** | ±0.58 |  | **6.16** | ±0.64 |  |
| FPRAU | **5.63** | ±0.89 |  | **5.97** | ±0.61 |  | **6.02** | ±0.71 |  | **5.93** | ±0.66 |  | **6.15** | ±0.41 |  | **5.91** | ±0.61 |  |
| DSV | **5.25** | ±0.84 |  | **5.28** | ±0.24 |  | **5.56** | ±0.62 |  | **5.47** | ±0.60 |  | **5.49** | ±0.29 |  | **5.54** | ±0.64 |  |
| CHIS | **5.25** | ±0.68 |  | **5.25** | ±0.33 |  | **5.51** | ±0.57 |  | **5.55** | ±0.60 |  | **5.53** | ±0.19 |  | **5.65** | ±0.58 |  |

| **Blank** | **t0** |  |  | **t4** |  |  | **t8** |  |  | **t24** |  |  | **t30** |  |  | **t48** |  |  |
| --- | --- | --- | --- | --- | --- | --- | --- | --- | --- | --- | --- | --- | --- | --- | --- | --- | --- | --- |
| EUB FL6 | **6.73** | ±0.65 |  | **6.95** | ±0.65 |  | **6.92** | ±0.52 |  | **6.78** | ±0.53 |  | **6.83** | ±0.16 |  | **7.03** | ±0.29 |  |
| BIF | **5.42** | ±0.68 |  | **5.85** | ±0.48 |  | **5.75** | ±0.27 |  | **5.63** | ±0.28 |  | **5.84** | ±0.22 |  | **5.94** | ±0.17 |  |
| LAB | **5.23** | ±0.80 |  | **5.41** | ±0.52 |  | **5.53** | ±0.41 |  | **5.96** | ±0.97 |  | **5.50** | ±0.16 |  | **5.81** | ±0.19 |  |
| BAC | **5.33** | ±0.71 |  | **5.73** | ±0.70 |  | **5.73** | ±0.55 |  | **5.62** | ±0.59 |  | **5.67** | ±0.24 |  | **6.00** | ±0.24 |  |
| EREC | **6.25** | ±0.66 |  | **6.21** | ±0.86 |  | **6.12** | ±0.65 |  | **5.80** | ±0.53 |  | **6.04** | ±0.44 |  | **6.01** | ±0.34 |  |
| RREC | **5.40** | ±0.48 |  | **5.67** | ±0.24 |  | **5.66** | ±0.27 |  | **5.46** | ±0.19 |  | **5.63** | ±0.09 |  | **5.92** | ±0.19 |  |
| ATO | **5.28** | ±0.47 |  | **5.56** | ±0.38 |  | **5.50** | ±0.29 |  | **5.67** | ±0.33 |  | **5.71** | ±0.14 |  | **5.90** | ±0.23 |  |
| PRO | **5.74** | ±0.69 |  | **6.07** | ±0.99 |  | **6.18** | ±0.92 |  | **5.72** | ±0.71 |  | **5.88** | ±0.32 |  | **6.13** | ±0.53 |  |
| FPRAU | **5.83** | ±0.86 |  | **5.93** | ±0.71 |  | **5.81** | ±0.54 |  | **5.69** | ±0.51 |  | **5.71** | ±0.26 |  | **5.95** | ±0.28 |  |
| DSV | **5.28** | ±0.78 |  | **5.60** | ±0.65 |  | **5.55** | ±0.36 |  | **5.38** | ±0.36 |  | **5.47** | ±0.05 |  | **5.84** | ±0.24 |  |
| CHIS | **5.24** | ±0.66 |  | **5.48** | ±0.45 |  | **5.52** | ±0.22 |  | **5.53** | ±0.55 |  | **5.54** | ±0.19 |  | **5.83** | ±0.23 |  |

**Figure 1:** Metabolite production for volunteers 2 (A) and 3 (B) with different substrates used at 1% of batch culture fermentation with 1% faecal slurry, which determined by GC-MS after 30 hours. Metabolites number are expressed as Mean±SD data (n=3). Graphs below is representing the metabolites for volunteer 2 and 3.

**Data of volunteer 3**

**Figure 2:** Concentration of SCFA from mixed culture fermentation supernatants at 30 hours using faecal innoculate from volunteer 2 and 3; High tyrosine (HT) (0.3:100 w/w), Low tyrosine (LT) (0.003:100 w/w), High tyrosine with FOS (HT with FOS) (0.3:100 w/w and 1.5:100 w/w), Low tyrosine with FOS (LT with FOS) (0.003:100 w/w and 1.5:100 w/w), Soybean (SB), Peptone meat extract (PM) and frucotooligosaccharide (FOS) (1.5:100 w/w) after 30 hrs incubation. The data presented as mean (±SEM) comparable to the control (*n*=3).

A= Donor 2, and B= Donor 3

A

B

**Figure 3:** Effect of different fermentation supernatants (High tyrosine (HT), High tyrosine with FOS (HT with FOS), Soybean (SB) and Peptone meat (PM) on DNA damage after 24 hrs exposure in the HT29 cell line. The data presented as mean (±SEM) percentage of DNA damage comparable to the control (n=3). * indicate a significant difference compared to the untreated control (Dunnett test; *p<0.05).

A= donor 2, and B= donor 3

A

*

**Comet data of volunteer 3**

B
